# Parliament and the Profession

**Published:** 1918-03-16

**Authors:** 


### PARLIAMENT AND THE PROFESSION.

### Army Dental Service.

Mr. Pennefather inquired of the Under-Secretary of State for War as to the position and prospects of qualified dental surgeons now serving in the ranks of the Army; and Mr. Macpherson informed him that the dental needs of the Army are not such that all qualified dentists serving can be used for their professional work.

### Royal Army Medical Corps.

A number of questions have been asked in the House of Commons lately as to when the Report on the Royal Army Medical Services will be made public. Mr. Macpherson recently informed Major Davies that the Army Council have not yet considered the Report, and it cannot be published until this stage has been reached. The recommendations are now being investigated by the British military authorities in France.

Dr. Macnamara stated that Royal Army Medical Corps officers are not under the Admiralty, who will arrange for Royal Naval medical officers to be lent to the Air Force on the completion of the medical arrangements for the Force.

The Under-Secretary of State for War informed Mr. Watt that R.A.M.C. officers serving on a yearly contract have a right to refuse to renew until they have had an opportunity of exercising their right of appeal to a tribunal.

### Physically Defective Children.

The President of the Board of Education informed Mr. Arnold Ward that twenty-five local education authorities have provided special schools for physically defective children. The system has been found satisfactory where adopted, and is certainly capable of useful extension in suitable areas.

